# Vaskulärer Verschluss nach Füllerinjektion mit Hyaluronsäure

**DOI:** 10.1007/s00105-020-04681-5

**Published:** 2020-09-16

**Authors:** Miriam Zidane, Lisa Eisert, Alexander Nast

**Affiliations:** 1 Klinik für Dermatologie, Venerologie und Allergologie, Charité-Universitätsmedizin Berlin, Berlin, Deutschland; 2 Klinik für Dermatologie und Venerologie, Vivantes Klinikum Neukölln, Berlin, Deutschland; 3 Klinik für Dermatologie, Venerologie und Allergologie, Division of Evidence-Based Medicine (dEBM), Charité – Universitätsmedizin Berlin, Charitéplatz 1, 10117 Berlin, Deutschland

## Anamnese

Eine 48-jährige Patientin stellte sich 4 Tage nach beidseitiger nasolabialer Injektion von Hyaluronsäure mit Schmerzen, Schwellung sowie Hautveränderungen links nasolabial in unserer Sprechstunde vor. Die Behandlung auf der linken Seite sei bereits während und direkt nach der Injektion deutlich schmerzhafter als auf der rechten Seite gewesen.

Einen Tag vor der Erstvorstellung in unserer Sprechstunde wurde durch den injizierenden Arzt eine antibiotische Therapie mit Cefuroxim 500 mg Tabletten 3‑mal täglich per os in Kombination mit Ibuprofen 600 mg Tabletten bei Bedarf eingeleitet. Darunter kam es zu einer weiteren Schmerzprogredienz und Befundverschlechterung. Die Patientin nahm keine weiteren Medikamente ein. Bis auf eine allergische Rhinitis lagen keine Vorerkrankungen vor.

## Klinischer Befund

**Figure 1:**
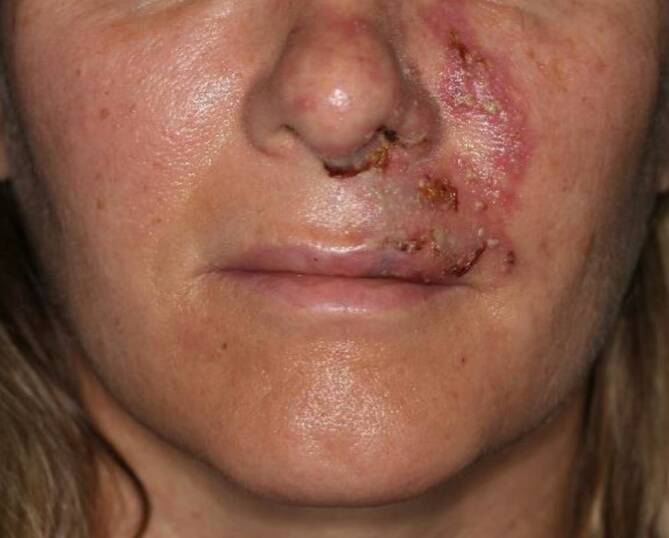

Bei Erstvorstellung in unserer Klinik zeigte sich eine rankenförmige, erythematös-violette, kissenartige Schwellung mit Pusteln im Bereich der Oberlippe, des Philtrums und der Wange links (Abb. 1).

## Labor

Laborchemisch wiesen wir ein erhöhtes C‑reaktives Protein (19,5 mg/l, Referenz: <5,0 mg/l) nach. Weitere Laborparameter einschließlich der Leukozyten waren ohne pathologischen Befund.

## Wie lautet die Verdachtsdiagnose?

In Zusammenschau von Anamnese und dem typischen klinischen Befund stellten wir den Verdacht auf einen vaskulären Verschluss nach Füllerinjektion mit Hyaluronsäure. Als mögliche Differenzialdiagnosen kommen ein Erysipel, eine Impetigo contagiosa und eine Herpesvirusinfektion in Betracht.

## Therapie

**Figure 2:**
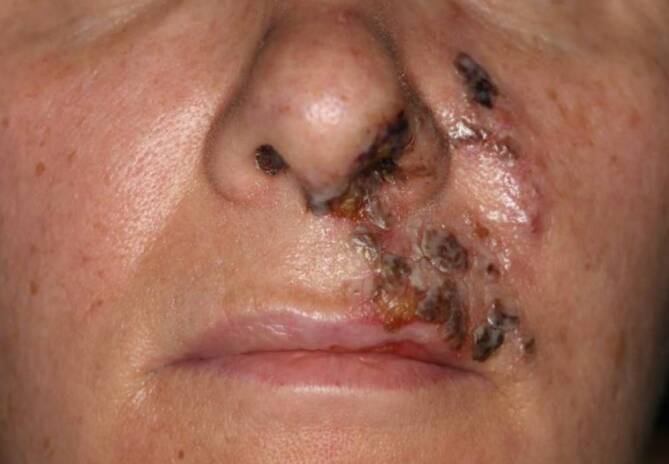

**Figure 3:**
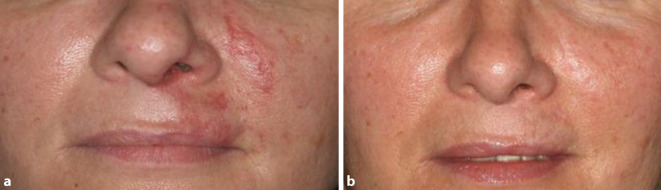

Zunächst erfolgte die Injektion von insgesamt 2 ml Hyaluronidase 150 IE/ml in Xylonest (Aspen Germany GmbH, München, Deutschland) läsional und periläsional. Zusätzlich wurden eine topische Therapie mit Linola® Sept Creme (Dr. August Wolff GmbH & Co. KG Arzneimittel, Bielefeld, Deutschland) sowie durchblutungsfördernde warme Umschläge eingeleitet. Fünf Tage nach Hyaluronidaseinjektion zeigte sich eine Abblassung des lividen Befundes mit Krustenbildung (Abb. 2). Die Schmerzen und Schwellung waren deutlich regredient. Die orale antibiotische Therapie wurde für weitere 10 Tage fortgeführt, die antiseptische Lokaltherapie behielten wir bei. Bei der Wiedervorstellung 28 Tage nach Hyaluronidaseinjektion konnten wir eine weitere Befundbesserung feststellen (Abb. 3). Neunzig Tage nach Hyaluronidaseinjektion entwickelte sich eine atrophe Narbe kranial der Oberlippe links (Abb. 3). Bei ausgeprägtem Behandlungswunsch der Patientin führten wir eine Weiterbehandlung der diskreten Narbenbildung mit dem fraktionierten CO_2_-Laser durch.

## Diskussion

Ein Gefäßverschluss bei Füllerinjektion mit Hyaluronsäure gehört zu den seltenen, aber nicht sicher vermeidbaren Komplikationen und hat einen beträchtlichen Einfluss auf die Lebensqualität von Patienten [2, 4]. Als „Antidot“ findet Hyaluronidase Einsatz [2, 3, 5]. Hyaluronidase spaltet komplexe Kohlenhydrate der extrazellulären Matrix und führt zu einer Hydrolyse von Hyaluronsäure und Mukopolysacchariden [1, 4]. Zugelassen ist Hyaluronidase in Kombination mit Lokalanästhetika zur Anwendung bei Injektionsanästhesietechniken (peribulbär, retrobulbär, „Sub-Tenon’s“) für ophthalmochirurgische Eingriffe. Es kommt zu einem schnelleren Wirkeintritt, Vergrößerung des schmerzunempfindlichen Bereiches und weniger intra- sowie postoperativen Schmerzen.

## Schlussfolgerung

Der Einsatz von Hyaluronidase bei Gefäßokklusion durch Füllerbehandlung mit Hyaluronsäure ist „off-label“. Besonders wichtig ist der zeitnahe Einsatz von Hyaluronidase, idealerweise innerhalb weniger Stunden [1]. Jeder Behandler und das weiter betreuende Praxispersonal sollten sensibilisiert sein für das „blanching“ im Ausbreitungsgebiet des entsprechenden Gefäßes und den plötzlich einsetzenden einseitigen Schmerz bei und direkt nach der Injektion [2]. In unserem Fall hätte spätestens bei der Wiedervorstellung der Patientin am Folgetag durch den klinischen Befund und die Schmerzen direkt nach der Injektion die Diagnose eines Gefäßverschlusses und die Behandlung mittels Hyaluronidase erfolgen sollen. Bis zu welchem Zeitpunkt nach Gefäßokklusion die Injektion von Hyaluronidase noch Erfolg versprechend ist, wurde bisher nicht systematisch untersucht. Bei unbedeutendem Risiko der Behandlung mit Hyaluronidase steht aus unserer Sicht jedoch der Nutzen auch Tage danach in einem vertretbaren Verhältnis. Es wird in der Regel eine Injektion sowohl direkt in das Injektionsareal als auch in die Umgebung empfohlen [2]. Jeder Behandler sollte über das Risiko und die Symptome eines Gefäßverschlusses aufklären, bei der Injektion aspirieren, in „kritischen Arealen“ wie den Nasolabialfalten ggf. Kanülen verwenden, langsam injizieren, bei „blanching“ und/oder einseitigen plötzlich auftretenden Schmerzen die Injektion stoppen und zur schnellen Intervention Hyaluronidase als „Notfallmedikament“ verfügbar haben [1, 4].

## Fazit für die Praxis

- Ein Gefäßverschluss bei Füllerinjektion mit Hyaluronsäure gehört zu den seltenen, aber nicht sicher vermeidbaren Komplikationen und hat einen beträchtlichen Einfluss auf die Lebensqualität von Patienten.
- Bei Gefäßokklusion durch Füllerbehandlung mit Hyaluronsäure ist der zeitnahe Einsatz von Hyaluronidase wichtig.
- Jeder Behandler und das weiter betreuende Praxispersonal sollten sensibilisiert sein für das „blanching“ im Ausbreitungsgebiet des entsprechenden Gefäßes und den plötzlich einsetzenden einseitigen Schmerz bei und direkt nach der Injektion.
